# Association Between Physical Activity Intervention Website Use and Physical Activity Levels Among Spanish-Speaking Latinas: Randomized Controlled Trial

**DOI:** 10.2196/13063

**Published:** 2019-07-24

**Authors:** Sarah E Linke, Shira I Dunsiger, Kim M Gans, Sheri J Hartman, Dori Pekmezi, Britta A Larsen, Andrea S Mendoza-Vasconez, Bess H Marcus

**Affiliations:** 1 Department of Family Medicine and Public Health University of California, San Diego La Jolla, CA United States; 2 Centers for Behavioral and Preventive Medicine, Department of Psychiatry and Human Behavior Miriam Hospital and Warren Alpert Medical School Brown University Providence, RI United States; 3 Department of Human Development and Family Studies Institute for Collaboration on Health, Intervention, and Policy University of Connecticut Storrs, CT United States; 4 Department of Behavioral and Social Sciences and the Institute for Community Health Promotion School of Public Health Brown University Providence, RI United States; 5 Department of Health Behavior School of Public Health University of Alabama Birmingham, AL United States

**Keywords:** physical activity, Latinas, internet, treatment engagement

## Abstract

**Background:**

The internet’s low cost and potential for high reach makes Web-based channels prime for delivering evidence-based physical activity (PA) interventions. Despite the well-studied success of internet-based PA interventions in primarily non-Hispanic white populations, evidence on Spanish-speaking Latinas’ use of such interventions is lacking. The recent rise in technology use among Latinas in the United States, a population at heightened risk for low PA levels and related conditions, suggests that they may benefit from Web-based PA interventions tailored to their cultural and language preferences.

**Objective:**

The goal of the research was to examine participant engagement with various features of an internet-based PA intervention for Latinas and explore how use of these features was differentially associated with adoption and maintenance of PA behavior change.

**Method:**

Pasos Hacia la Salud tested a Spanish-language, culturally adapted, individually tailored, internet-based PA intervention versus a Spanish language, internet-based, Wellness Contact Control condition for underactive Latinas (N=205, mean age 39.2 [SD 10.5] years, 84% Mexican American). These analyses examined engagement with the website and explored how use was associated with adoption and maintenance of moderate to vigorous physical activity (MVPA) behavior.

**Results:**

Overall, participants logged on to the website an average of 22 times (SD 28) over 12 months, with intervention participants logging on significantly more than controls (29 vs 14.7, *P*<.001). On average, participants spent more time on the website at months 1, 4, and 6 compared to all other months, with maximum use at month 4. Both log-ins and time spent on the website were significantly related to intervention success (achieving higher mean minutes of MVPA per week at follow-up: *b*=.48, SE 0.20, *P*=.02 for objectively measured MVPA and *b*=.74, SE 0.34, *P*=.03 for self-reported MVPA at 12 months, controlling for baseline). Furthermore, those meeting guidelines by the Centers for Disease Control and Prevention for PA at 12 months (≥150 minutes per week of MVPA) logged on significantly more than those not meeting guidelines (35 vs 20 over 12 months, *P*=.002). Among participants in the intervention arm, goal-setting features, personal PA reports, and PA tips were the most used portions of the website. Higher use of these features was associated with greater success in the program (significantly more minutes of self-reported MVPA at 12 months controlling for baseline). Specifically, one additional use of these features per month over 12 months translated into an additional 34 minutes per week of MVPA (goals feature), 12 minutes per week (PA tips), and 42 minutes per week (PA reports).

**Conclusions:**

These results demonstrate that greater use of a tailored, Web-based PA intervention, particularly certain features on the site, was significantly related to increased PA levels in Latinas.

**Trial Registration:**

ClinicalTrials.gov NCT01834287; https://clinicaltrials.gov/ct2/show/NCT01834287

## Introduction

Robust evidence supports physical activity (PA) in the prevention and management of numerous chronic health conditions, including obesity, hypertension, type 2 diabetes, cardiovascular diseases, and many types of cancer [1]. Despite these well-established benefits of regular PA, most Americans are insufficiently active [2]. Interventions designed to promote PA have had mixed success, and even those that have resulted in overall increases in PA have shown a range of adherence to behavior change among individuals [3]. A better understanding of the intervention features that successfully promote PA can inform the development of more efficient and broadly effective interventions.

Treatment engagement is a consistent predictor of success in health behavior interventions [4-6]. It is analogous to taking medication as prescribed; if the proper dose is not taken, the medication will be less effective. One of the greatest challenges facing behavioral scientists, therefore, is designing interventions to optimize treatment engagement and adherence and, in effect, maximize efficacy [7]. Delivering intervention materials and information in convenient and efficient ways may help to increase their uptake.

Use of technology, such as the Web, could be a promising solution for increasing engagement. Internet-based interventions offer numerous benefits for researchers and participants such as convenience, portability, adaptability, cost effectiveness, and reach. Internet-based interventions can incorporate multiple behavioral adherence strategies such as self-monitoring, social support, and goal-setting in a more accessible manner than in-person or print-based health behavior change interventions, and the interactive features of websites could promote participant engagement [4]. Additionally, internet-based interventions allow for objective measures of participant engagement by tracking variables such as number of log-ins, page clicks, and time spent on various components of the intervention. As internet-based interventions have achieved mixed success (ie, some are successful and others are not) [8], a more thorough examination of these objective measures of engagement could be especially useful in illuminating which intervention features promote not only engagement in the intervention but also successful behavior change.

In addition to promoting and tracking participant engagement, internet-based interventions could be an especially effective delivery channel for racial and ethnic minorities. Internet use in Latinos has grown markedly in recent years [9]. In 2015, 84% of Latinos were online with the fastest growing use among immigrant Hispanics and those who are Spanish dominant [10]. The Latino population also reports lower levels of PA than non-Latino whites [11]. Latina women report less activity than non-Latino white women and Latino men, and Latinas also experience higher rates of obesity, diabetes, and other chronic conditions related to inactive lifestyle [11]. Given the rise in internet use among Latinas and the potential of internet-based interventions to incorporate features associated with successful behavior change, internet-based interventions may be particularly appropriate for promoting PA in Latinas.

The purpose of this paper was to examine participant engagement with various features of an internet-based PA intervention for Latinas and explore how use of these features was differentially associated with adoption and maintenance of PA behavior change.

## Methods

### Overview of Trial

The Pasos Hacia La Salud study was a randomized controlled trial of an internet-based PA intervention versus a wellness contact control in Latinas (N=205). Engagement data were collected between 2011 and 2014 and included information on website use (number of log-ins, time spent on websites, and number of times PA goals were set, personal PA reports were accessed, and PA tips features were used throughout the project period). Minutes of moderate to vigorous physical activity (MVPA) were measured subjectively and objectively at baseline, 6 months, and 12 months.

### Setting and Sample

The study was conducted at the University of California, San Diego, and human subjects approval was obtained from the institutional review board. The study was registered as a clinical trial [NCT01834287]. Participants in the trial included underactive Latinas (defined as participating in MVPA less than 60 minutes per week) aged between 18 and 65 years with regular internet access through home, work, or their community. Individuals were excluded from participation if they had any serious medical condition that would make unsupervised PA unsafe or were unable to read or speak Spanish fluently and demonstrate adequate functional health literacy (scoring at least a 17 on the Short Test of Functional Health Literacy in Adults [12]; currently pregnant or planning to become pregnant in the next year; planning to move from the area within the next year; hospitalized due to a psychiatric disorder in the past 3 years; or taking medication that may impair PA tolerance or performance [13]. The setting, sample, and primary outcomes are described in further detail in previously published manuscripts [13,14].

### Protocol

Participants were screened for initial eligibility by phone and then completed baseline PA assessments before being randomly assigned to one of two Spanish language internet-based conditions: Tailored Physical Activity Internet Intervention or Wellness Contact Control Internet Intervention. A detailed description of study protocols can be found elsewhere [13]. At the baseline randomization visit, research staff explained the intervention and participant expectations and helped intervention arm participants set realistic PA goals, identify potential barriers and potential solutions, and learn to use website features.

#### Tailored Physical Activity Internet Intervention

The Tailored Physical Activity Internet Intervention was based on the transtheoretical model [15] and social cognitive theory [16]. Participants in this arm received access to the intervention website and completed monthly online surveys that generated automated tailored PA feedback reports on relevant theoretical constructs such as current stage of motivational readiness for PA, self-efficacy, and processes of change, as well as normative and progress feedback (ie, how they compared on these variables vs others who were already meeting guidelines and to their own prior responses) and useful facts on PA health benefits, stretching, and heart rate monitoring. The reports drew from a bank of more than 300 messages addressing different levels of these psychosocial and environmental factors affecting PA. In addition, participants received an online manual that was matched to their motivational readiness for PA. The manual emphasized strategies for increasing PA such as goal-setting, self-monitoring, problem-solving barriers, methods for increasing social support, and rewarding oneself for meeting PA goals.

Additional website features included (1) self-monitoring of minutes of PA and steps, (2) goal setting with graphs to compare goals to recorded minutes, (3) a message board to foster social support between participants, (4) an “ask the expert” section where participants could anonymously ask questions to a PhD-level researcher, and (5) online resources such as free exercise videos and maps to create walking routes. The intervention group received email prompts to access the intervention website weekly during month 1, biweekly during months 2 and 3, and monthly during months 4 to 6, with new PA information tip sheets made available on this schedule. Participants received monetary incentives to complete the study requirements, including $10 each month for completing the online monthly questionnaires. They also received a pedometer to track their steps (although minutes of MVPA rather than step count was the outcome of interest).

#### Wellness Contact Control Internet Group

The Wellness Contact Control Internet Group received access to a Spanish language website with information on health topics other than PA. The Web-based content focused on diet and other factors associated with cardiovascular disease risk and included information from a series on heart health developed for Latinos by the National Heart Lung and Blood Institute. Control arm participants received the same number of email contacts on the same schedule as the intervention arm and also completed monthly surveys on wellness topics for the same $10 incentive offered to intervention arm participants.

### Measures

Demographics were assessed at baseline with a brief questionnaire assessing age, education, income, occupation, race, ethnicity, history of residence in the United States, marital status, and acculturation.

PA was measured subjectively using the 7-Day Physical Activity Recall (7-Day PAR) and objectively using accelerometers. The 7-Day PAR is an interviewer-administered instrument that provides an estimate of weekly minutes of PA and has consistently demonstrated acceptable reliability, internal consistency, and concurrent validity with objective measures of activity [17-19]. Accelerometers measure both movement and intensity of activity and have been validated with heart rate telemetry [20] and total energy expenditure [21]. Participants were asked to wear the accelerometer on their left hip for 7 days. Valid wear time was classified as 5 days of at least 600 minutes of wear time each day or at least 3000 minutes of wear time over 4 days. To be counted in the total minutes per week of MVPA, activity had to occur in ≥10-minute bouts, per the national PA guidelines at the time of the study. Accelerometer data was processed using the ActiLife software, with the established cut point of 1952 counts per minute to meet the minimum threshold for moderate intensity activity [22].

Website use was tracked throughout the project period using built-in software. Variables of interest included number of log-ins and time spent on websites for all participants, as well as the number of times key intervention website features related to PA goal-setting, personal PA reports, PA tips, and message board for social support were used. Self-reported satisfaction with the website was measured using consumer satisfaction questions on the follow-up surveys.

### Data Analysis

Overall log-ins to the study website were summarized monthly and compared between groups at each month using *t* tests. In addition, changes over time in number of log-ins within group was compared using a generalized linear model. Total time spent on the website was calculated for each arm during the intervention period (baseline to 6 months), maintenance period (6 to 12 months), and total study period (baseline to 12 months). Using a series of generalized linear models, we tested the effect of total time spent on the website and total number of log-ins over 12 months on both primary measures of intervention success (minutes per week of MVPA collected subjectively via the 7-day PAR and objectively via accelerometer). Models were adjusted for baseline MVPA, group, and wear time (in the case of accelerometry).

As a secondary outcome, we examined the effects of time spent on the website and number of log-ins on the odds of meeting national American College of Sports Medicine (ACSM) guidelines [23] for MVPA (≥150 minutes per week) at 12 months, measured subjectively and objectively.

Using a series of generalized linear models, we tested the association between time using each feature and PA outcomes (self-report and objectively measured minutes per week of MVPA) using a series of univariate models. Significant features (defined as those for which increases in use were significantly associated with increased minutes of MVPA at 12 months controlling for baseline) were then considered as part of a multivariate model predicting 12-month outcomes.

Associations between log-ins and targeted psychosocial constructs during the adoption phase were explored using a series of generalized linear models in which the mean value of the construct (eg, self-efficacy) at 6 months (primary end point) was regressed on baseline value of the construct, number of log-ins over 6 months, and treatment arm.

Finally, self-reported satisfaction with the website was summarized, and descriptive statistics are reported for the intervention arm. All analyses were carried out in SAS 9.3 (SAS Institute), with significance level set at alpha=.05 a priori.

## Results

Participants (N=205) were all Latina, mostly Mexican American (172/205, 83.9%), with an average age of 39.2 (SD 10.5) years. Full descriptions of participant demographics are presented in Table 1.

### Physical Activity Outcomes

Main outcomes of self-reported and objectively measured minutes of MVPA in both groups across all time points are reported in Table 2. Intervention arm participants demonstrated significantly greater gains in MPVA than control arm participants. Intervention participants were more likely to report meeting ACSM guidelines (≥150 minutes per week of MVPA) than control participants at 6 (31% vs 12%) and 12 (29% vs 19%) months, although group differences were not significant with accelerometer data at 6 (13% vs 9%) or 12 (16% vs 13%) months.

**Table 1:** Demographic characteristics.

Characteristics		Intervention (n=104)	Control (n=101)
Hispanic, n (%)		104 (100)	101 (100)
Age (years), mean (SD)		38.8 (10.6)	39.6 (10.4)
First generation in the United States, n (%)		90 (86.5)	78 (77.2)
Body mass index (kg/m^2^), mean (SD)		29.1 (5.8)	28.6 (4.5)
**Race, n (%)**			
	White	47 (45.2)	59 (58.4)
	Mixed	18 (17.3)	15 (14.9)
	Other	32 (30.8)	19 (18.8)
**Ethnicity, n (%)**			
	Mexican	86 (82.7)	87 (86.1)
	Columbian	2 (1.9)	5 (5.0)
	Guatemalan	2 (1.9)	0 (0)
	Puerto Rican	1 (1.0)	1 (1.0)
	Dominican Republic	1 (1.0)	0 (0)
	Other	15 (14.4)	11 (10.9)
**Yearly household income, n (%)**			
	Less than $30,000	72 (69.3)	64 (63.5)
	$30,000-$50,000	18 (17.3)	25 (24.7)
	$50,000 or more	10 (9.6)	7 (6.9)
	Don’t know	4 (3.8)	5 (5.0)
**Employment status, n (%)**			
	Unemployed	51 (49.0)	41 (41.0)
	Part-time	26 (25.0)	30 (30.0)
	Full-time	26 (25.0)	29 (29.0)
	Refused/no answer	1 (1.0)	0 (0)
**Education level, n (%)**			
	Less than high school	15 (14.6)	14 (13.9)
	High school graduate	16 (15.5)	8 (7.9)
	Vocational/technical	15 (14.6)	12 (11.9)
	Some college	57 (55.4)	67 (66.3)
**Language spoken in home, n (%)**			
	Only Spanish	42 (40.4)	35 (34.7)
	More Spanish	32 (30.8)	33 (32.7)
	Both equally	16 (15.4)	24 (23.8)
	More English	12 (11.5)	5 (5.0)
	Only English	2 (1.9)	4 (4.0)
**Marital status, n (%)**			
	Married	52 (50.0)	58 (57.4)
	Living with partner	5 (4.8)	6 (5.9)
	Separated	14 (13.5)	3 (3.0)
	Divorced	11 (10.6)	17 (16.8)
	Widowed	2 (1.9)	3 (3.0)
	Never married	20 (19.2)	14 (13.9)
Health literacy score (score of 23-26 adequate)		34.8 (2.7)	37.2 (22.8)

**Table 2:** Self-reported and objective moderate to vigorous physical activity at each study time point.

Variable	Intervention, mean (SD)			Control, mean (SD)		
	Baseline	6 months	12 months	Baseline	6 months	12 months
Self-reported MVPA^a^ (min/wk), 7-day PAR^b^, (n=205)	8.0 (15.0)	112.8 (97.1)	108.6 (107.2)	10.4 (24.0)	63.0 (88.3)	75.9 (89.8)
Accelerometer measured MVPA in 10-min bouts (min/wk), (n=200)	35.8 (69.7)	75.8 (91.0)	70.4 (86.4)	28.7 (48.2)	43.0 (60.9)	55.5 (74.6)

^a^MVPA: moderate to vigorous physical activity.

^b^PAR: physical activity recall.

### Treatment Engagement

**Figure 1:**
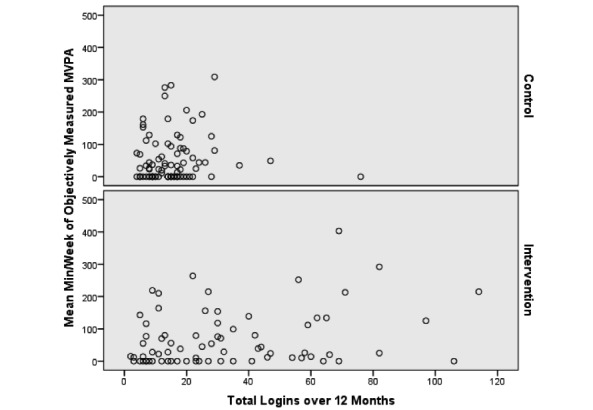
Unadjusted association between log-ins and self-reported moderate to vigorous physical activity over 12 months.

**Figure 2:**
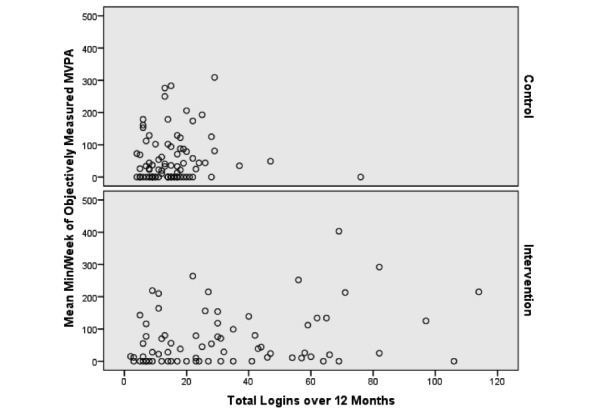
Unadjusted association between log-ins and objectively measured moderate to vigorous physical activity over 12 months.

Overall, participants logged in to the website an average of 22 (SD 28) times over 12 months, with intervention participants logging on significantly more than controls (29 vs 14.7, *P*<.001). On average, intervention participants spent more time on the website at months 1, 4, and 6 compared to all other months, with maximum use at month 4. Unadjusted associations between log-ins and self-reported MVPA are presented in Figure 1. Unadjusted associations between log-ins and objectively measured MVPA are presented in Figure 2.

**Figure 3:**
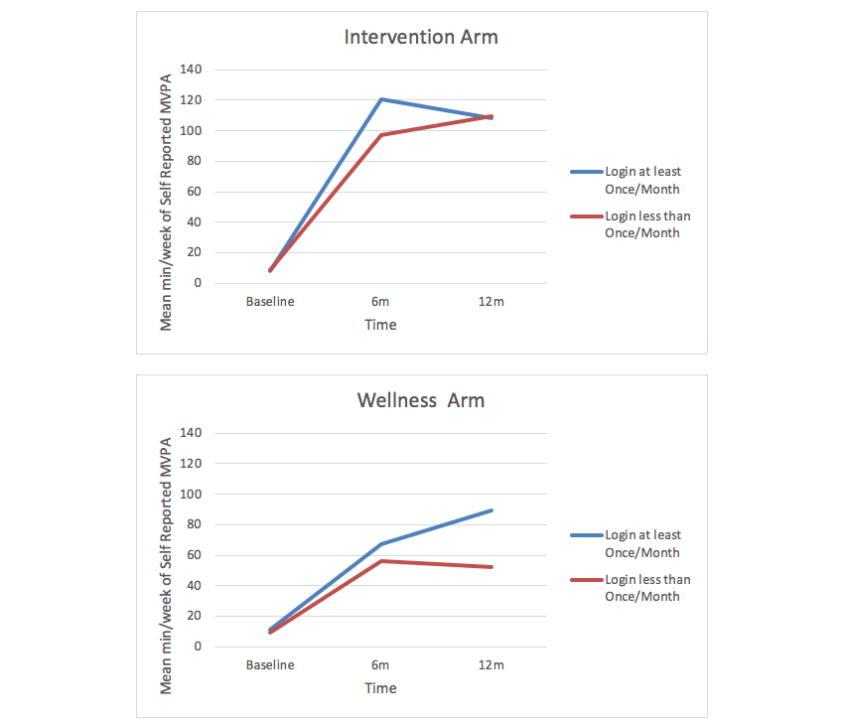
Comparing log-in rates and moderate to vigorous physical activity over time by group.

Both log-ins and time spent on the website were significantly related to intervention success (achieving higher mean minutes of MVPA per week at 12-month follow-up, controlling for baseline: *b*=.48, SE 0.20, *P*=.02 for objectively measured MVPA and *b*=.74, SE 0.34, *P*=.03 for self-reported MVPA). Furthermore, those meeting ACSM guidelines for PA at 12 months (≥150 minutes per week of self-reported MVPA) spent significantly more time on the website than those not meeting guidelines (35 vs 20 minutes over 12 months, *P*=.002). Figure 3 illustrates differences in the association between log-ins and self-reported MVPA by group over time. A significant difference in self-reported MVPA was apparent at 6 months for participants who logged in at least 1 time per month in the intervention arm (*P*=.04) but not the control arm (*P*=.54). At the 12-month follow-up, no difference was apparent in the intervention arm (*P*=.94), but the difference approached significance in the control arm (*P*=.054).

Among participants in the intervention arm, goal-setting features, personal PA reports, and PA tips were the most used portions of the website. Higher use of these features was associated with greater success in the program (more minutes of self-reported MVPA at 12 months controlling for baseline). Specifically, one additional use of these features per month over 12 months translated into an additional 34 minutes per week of MVPA (goals feature), 12 minutes per week (PA tips), and 42 minutes per week (PA reports). Results of the adjusted analyses are presented in Table 3.

Exploratory analyses suggest that independent of the effect of randomization, number of log-ins was positively associated with greater self-efficacy (*b*=.004, SE 0.002, *P*=.05) and social support (rewards and punishment subscale: *b*=.006, SE 0.003, *P*=.03) at 6 months such that more log-ins was associated with higher 6 months scores on these variables controlling for baseline. There were no significant effects of log-ins on psychosocial constructs (behavioral and cognitive processes, enjoyment, and social support family and friends scores) at 6 months.

**Table 3:** Adjusted associations between website components and self-reported moderate to vigorous physical activity at 12 months among intervention participants.

Component	*b*^a^	SE	*P* value
Goal setting	2.85	1.38	.04
Physical activity tips	1.00	0.82	.05
Physical activity reports	3.49	1.28	.008

^a^*b*: unstandardized regression coefficient. Models adjusted for baseline physical activity.

### Consumer Satisfaction Survey

All participants in the intervention arm reported that the intervention was at least somewhat enjoyable (66/66, 100%), and the majority (62/66, 94%) reported that the website was at least somewhat motivating. Ease of use of the website features was rated on a 5-point scale ranging from not at all easy (0) to very easy (5). The majority of participants reported that the following features were at least somewhat easy to use: recording activity (60/67, 90%), setting goals (59/67, 88%), message board (54/67, 81%), ask the expert (50/67, 75%), and places to be active (38/67, 57%). Less than half of participants (32/67, 48%) reported the MapMyWalk feature to be somewhat or very easy to use. Most participants reported using most of the features, including the self-monitoring/activity record (58/64, 91%), goal setting 58/64, 91%), message board (52/63, 83%), ask an expert (47/63, 75%), places to be active (37/63, 59%), and MapMyWalk (31/63, 49%). However, when asked to rank the most helpful feature of the website, the most commonly first ranked feature was MapMyWalk (10/63, 16%), followed by online exercise resources (6/63, 10%). The majority of participants reported gaining at least some knowledge from the website (64/66, 97%), and most (62/65, 95%) reported that the monthly email questionnaire reminders were at least somewhat helpful.

## Discussion

### Principal Findings

These results demonstrate that greater use of a tailored, internet-based PA intervention, particularly certain features on the website, was significantly related to increased PA levels in Latinas. These positive dose-response results support the existing treatment engagement literature [24-26] and suggest that PA and other health behavior interventions may be more successful than they appear in intent-to-treat analyses, which include all participants regardless of the actual dose they receive of the intervention. The results from this study are encouraging for health promotion researchers in that they demonstrate that an evidence-based internet-delivered intervention might have higher efficacy when used as intended. Given that lack of engagement is a major obstacle in health promotion research [27], these findings also reveal a need to identify effective strategies to promote engagement and treatment adherence.

Although participants of the Pasos Hacia La Salud intervention reported using most of the intervention features, our analyses of user data revealed that some features were more frequently used than others and that the level of engagement of participants with different intervention features had an effect on the number of minutes per week of MVPA. Engagement in internet-based health promotion interventions may be subject to unique challenges, driven mainly by limited face-to-face contact with participants [27,28]. Internet-based interventions, however, also grant unique opportunities for the implementation of strategies that have been associated with increased engagement, such as tailoring and personalization of messages [26,29,30]. In the Pasos Hacia La Salud intervention, tailoring and personalization were successfully employed in different website elements, including goal-setting and personalized reports; both of these intervention components were associated with the greatest increases in MVPA. On the other hand, increased use of other website features, including the possibility to interact with the researcher through the website and the group chat (meant to provide social support), was not associated with increased MVPA. In previous research, social support has produced mixed results for engagement, with some studies, but not all, showing increased support to be associated with increased engagement and adherence [30-32]. One study found that an online community increased intervention adherence and those who had little support at baseline used and benefited more from this feature compared with those who already had a supportive social network [31]. Perhaps a limitation of the message board feature on our website was that participants were anonymous and did not know each other, so they may not have been motivated to interact with each other or depend on each other for social support. Future research could consider including a social media component as part of the online intervention.

This study also revealed that overall engagement with the website peaked at key study time points (ie, around measurement and intervention visits) but overall decreased over time, which is also consistent with previous literature [24,25]. Moreover, the effect of increased engagement among intervention participants (specifically, logging in at least once per month) on minutes per week of MVPA seems to have dissipated during the maintenance period, as shown in Figure 3, perhaps as a result of this decreased engagement over time. A number of factors may help explain this decline in engagement. For example, participants may have become increasingly independent in their behavior and decreasingly dependent on tools available through the website or they may have become bored with the relatively static content despite regular updates such as daily PA tips and message board discussions. Another potential explanation is that these results may reflect the limited use of prompts and email reminders sent to participants during the tapered maintenance period (months 6 to 12) compared to the active intervention period (months 1 to 6), as previous research suggests that reminders are important to maintain engagement among intervention participants [32]. Curiously, the relationship between monthly log-ins and MVPA minutes in the control group grew stronger at 12 months, nearing statistical significance. Unfortunately we do not have an explanation for this unexpected differential finding. Identifying strategies for continuous engagement with behavior change tools during maintenance stages is an important area for future research. Text messages would be a possible intervention to explore.

Another important research question is to examine whether individuals and populations who are less tech-savvy are less likely to remain engaged with the different features of technology-based interventions. For example, it is notable that the feature MapMyWalk was ranked the most helpful by many intervention participants but was also seen as a less user-friendly feature by approximately half of the participants. Although this feature might have filled a specific need among those who were able to use it, increased user-friendliness might have enabled more participants to benefit from it. Thus, it is important to identify strategies to simplify this and similar intervention features to make them more accessible to participants who may be less tech-savvy in order to promote increased engagement and ensuing behavior change. Furthermore, future research should test interface design features that might better engage Latino populations so they are more likely to access and effectively use internet-based interventions for improving physical activity and other health behaviors. For example, future research could include delivery of internet- or app-based interventions that can be accessed on mobile phones rather than computers, as Latinos are heavily reliant on mobile phones for their internet access, more than other ethnicities. While Latinos have lagged other groups in accessing the internet and having broadband at home, they have been among the most likely to own a mobile phone and access the internet from a mobile device.

The results of this study are particularly encouraging for researchers focusing on Latino populations, who face a higher risk of chronic diseases and other health problems related to insufficient PA. Linguistically and culturally adapting existing evidence-based treatment materials to match the needs of this underserved population and delivering them via the internet appears to be a feasible way to reach this population. Overall, the majority of intervention participants reported some knowledge gain and found the intervention to be at least somewhat enjoyable and engaging. Additionally, successfully engaging participants in online tools to address behavior change techniques appears to result in significant increases in PA. Moreover, the frequency and time necessary for these significant gains was relatively minimal, suggesting that the participant burden was low and the cost was effective.

### Limitations

This study has certain limitations. Mainly, adherence and engagement cannot be experimentally tested, and thus we cannot rule out confounding variables driving the observed relationships. Other factors (eg, self-efficacy, motivation to change) may be driving both engagement and changes in MVPA. Additionally, although we have objective measures of engagement, such as number of log-ins and time spent on the website, these measures may be susceptible to error. For example, time spent on the website may be imprecise if participants remained logged into the website for a period of time but did not engage with it throughout the entire period (for example, if they were browsing other websites while remaining logged in to the study website). Nevertheless, by using two different objective measures of engagement (log-ins and time spent), as opposed to solely one measure, we aimed to corroborate the results and strengthen the evidence. Another limitation is that a component of the study intervention was emailing participants to remind them to log in and complete their monthly questionnaire in order to receive a $10 incentive, which may have differentially influenced the amount of user engagement with different features of the website, specifically encouraging use of the personalized reports feature. However, it may have also served to drive participants to the website when they would not have otherwise logged in at all and increased log-ins and time spent on multiple features of the website. As such incentives may not be replicable in dissemination of Web-based interventions, future research should examine the role and importance of incentives in website engagement. Monetary and nonmonetary (eg, attention, social support) incentives to participate in this study are not scalable in real-world settings, and thus the generalizability of the results from a public health standpoint should be tempered.

### Conclusions

Overall, results of this study suggested that greater use of an internet-based PA intervention, particularly certain features of the website, was significantly related to increased PA levels in Latinas. These results are encouraging from a public health perspective because this type of intervention can be delivered on a large scale at a relatively low cost once it is developed and published online. Because data suggests that Latinas are using the internet and other technology-based devices at a rapid pace, developing and disseminating these types of interventions is a potentially cost-effective way to help address the PA-related health disparities faced by this population. These findings also emphasize the importance of identifying effective ways to promote engagement and adherence to specific components to help ensure that participants receive the intended dose of the intervention.

## Abbreviations

PA: physical activity
MVPA: moderate to vigorous physical activity
PAR: Physical Activity Recall
ACSM: American College of Sports Medicine

## Appendix

Multimedia Appendix 1 CONSORT‐EHEALTH checklist (V 1.6.1).

## References

[1] Warburton DER, Nicol CW, Bredin SSD (2006). Health benefits of physical activity: the evidence. CMAJ.

[2] Schiller JS, Lucas JW, Ward BW, Peregoy JA (2012). Summary health statistics for U.S. adults: National Health Interview Survey, 2010. Vital Health Stat 10.

[3] Greaves CJ, Sheppard KE, Abraham C, Hardeman W, Roden M, Evans PH, Schwarz P, IMAGE Study Group (2011). Systematic review of reviews of intervention components associated with increased effectiveness in dietary and physical activity interventions. BMC Public Health.

[4] Vandelanotte C, Spathonis KM, Eakin EG, Owen N (2007). Website-delivered physical activity interventions a review of the literature. Am J Prev Med.

[5] Jelalian E, Hart CN, Mehlenbeck RS, Lloyd-Richardson EE, Kaplan JD, Flynn-O'Brien KT, Wing RR (2008). Predictors of attrition and weight loss in an adolescent weight control program. Obesity (Silver Spring).

[6] Hardcastle S, Blake N, Hagger MS (2012). The effectiveness of a motivational interviewing primary-care based intervention on physical activity and predictors of change in a disadvantaged community. J Behav Med.

[7] Leslie E, Marshall AL, Owen N, Bauman A (2005). Engagement and retention of participants in a physical activity website. Prev Med.

[8] Norman GJ, Zabinski MF, Adams MA, Rosenberg DE, Yaroch AL, Atienza AA (2007). A review of eHealth interventions for physical activity and dietary behavior change. Am J Prev Med.

[9] Lopez M, Gonzalez-Barrera A, Patten E (2013). Closing the digital divide: Latinos and technology adoption.

[10] Brown A, López G, Lopez M (2016). Digital divide narrows for Latinos as more Spanish speakers and immigrants go online.

[11] Blackwell DL, Lucas JW, Clarke TC (2014). Summary health statistics for U.S. adults: National Health Interview Survey, 2012. Vital Health Stat 10.

[12] Baker DW, Williams MV, Parker RM, Gazmararian JA, Nurss J (1999). Development of a brief test to measure functional health literacy. Patient Educ Couns.

[13] Marcus BH, Hartman SJ, Pekmezi D, Dunsiger SI, Linke S, Marquez B, Gans KM, Bock BC, Larsen BA, Rojas C (2015). Using interactive Internet technology to promote physical activity in Latinas: Rationale, design, and baseline findings of Pasos Hacia La Salud. Contemp Clin Trials.

[14] Marcus BH, Hartman SJ, Larsen BA, Pekmezi D, Dunsiger SI, Linke S, Marquez B, Gans KM, Bock BC, Mendoza-Vasconez AS, Noble ML, Rojas C (2016). Pasos Hacia La Salud: a randomized controlled trial of an internet-delivered physical activity intervention for Latinas. Int J Behav Nutr Phys Act.

[15] Marcus BH, Selby VC, Niaura RS, Rossi JS (1992). Self-efficacy and the stages of exercise behavior change. Res Q Exerc Sport.

[16] Bandura A (1986). Social Foundations of Thought and Action: A Social Cognitive Theory.

[17] Richardson MT, Ainsworth BE, Jacobs DR, Leon AS (2001). Validation of the Stanford 7-day recall to assess habitual physical activity. Ann Epidemiol.

[18] Sallis JF, Haskell WL, Wood PD, Fortmann SP, Rogers T, Blair SN, Paffenbarger RS (1985). Physical activity assessment methodology in the Five-City Project. Am J Epidemiol.

[19] Hayden-Wade HA, Coleman KJ, Sallis JF, Armstrong C (2003). Validation of the telephone and in-person interview versions of the 7-day PAR. Med Sci Sports Exerc.

[20] Janz KF (1994). Validation of the CSA accelerometer for assessing children's physical activity. Med Sci Sports Exerc.

[21] Melanson EL, Freedson PS (1995). Validity of the Computer Science and Applications Inc (CSA) activity monitor. Med Sci Sports Exerc.

[22] Freedson PS, Melanson E, Sirard J (1998). Calibration of the Computer Science and Applications Inc accelerometer. Med Sci Sports Exerc.

[23] Garber CE, Blissmer B, Deschenes MR, Franklin BA, Lamonte MJ, Lee I, Nieman DC, Swain DP (2011). American College of Sports Medicine position stand. Quantity and quality of exercise for developing and maintaining cardiorespiratory, musculoskeletal, and neuromotor fitness in apparently healthy adults: guidance for prescribing exercise. Med Sci Sports Exerc.

[24] Cugelman B, Thelwall M, Dawes P (2011). Online interventions for social marketing health behavior change campaigns: a meta-analysis of psychological architectures and adherence factors. J Med Internet Res.

[25] Glasgow RE, Christiansen SM, Kurz D, King DK, Woolley T, Faber AJ, Estabrooks PA, Strycker L, Toobert D, Dickman J (2011). Engagement in a diabetes self-management website: usage patterns and generalizability of program use. J Med Internet Res.

[26] Strecher VJ, McClure J, Alexander G, Chakraborty B, Nair V, Konkel J, Greene S, Couper M, Carlier C, Wiese C, Little R, Pomerleau C, Pomerleau O (2008). The role of engagement in a tailored web-based smoking cessation program: randomized controlled trial. J Med Internet Res.

[27] Vandelanotte C, Müller AM, Short CE, Hingle M, Nathan N, Williams SL, Lopez ML, Parekh S, Maher CA (2016). Past, present, and future of eHealth and mHealth research to improve physical activity and dietary behaviors. J Nutr Educ Behav.

[28] Leslie E, Marshall AL, Owen N, Bauman A (2005). Engagement and retention of participants in a physical activity website. Prev Med.

[29] Morrison L, Moss-Morris R, Michie S, Yardley L (2014). Optimizing engagement with Internet-based health behaviour change interventions: comparison of self-assessment with and without tailored feedback using a mixed methods approach. Br J Health Psychol.

[30] Redfern J, Santo K, Coorey G, Thakkar J, Hackett M, Thiagalingam A, Chow CK (2016). Factors influencing engagement, perceived usefulness and behavioral mechanisms associated with a text message support program. PLoS One.

[31] Richardson CR, Buis LR, Janney AW, Goodrich DE, Sen A, Hess ML, Mehari KS, Fortlage LA, Resnick PJ, Zikmund-Fisher BJ, Strecher VJ, Piette JD (2010). An online community improves adherence in an internet-mediated walking program. Part 1: results of a randomized controlled trial. J Med Internet Res.

[32] Kelders SM, Kok RN, Ossebaard HC, Van Gemert-Pijnen J (2012). Persuasive system design does matter: a systematic review of adherence to web-based interventions. J Med Internet Res.

